# Incidence of Intrapartum-Related Events at the Largest Obstetric Hospital in Hanoi, Vietnam: A Retrospective Study

**DOI:** 10.3390/children9030321

**Published:** 2022-02-28

**Authors:** Tina Dempsey, Huong Lien Nguyen, Huong Thu Nguyen, Xuan Anh Bui, Phuong Thi Thu Pham, Toan K. Nguyen, Francesco Cavallin, Daniele Trevisanuto, Susanna Myrnerts Höök, Nicolas Pejovic, Mats Blennow, Linus Olson, Hien Vu, Anh Duy Nguyen, Tobias Alfvén

**Affiliations:** 1 Department of Global Public Health, Karolinska Institutet, 17177 Solna, Sweden; susanna.myrnerts.hook@ki.se (S.M.H.); nicolas.pejovic@ki.se (N.P.); linus.olson@ki.se (L.O.); tobias.alfven@ki.se (T.A.); 2 Astrid Lindgren Children’s Hospital, Karolinska University Hospital, 17176 Solna, Sweden; 3 Neonatal Department, Phu San Hanoi Hospital, Hanoi 100000, Vietnam; lienhuongnguyenhmu@gmail.com (H.L.N.); nguyenthuhuongkh90@gmail.com (H.T.N.); bsphuong.1971@gmail.com (P.T.T.P.); 4 Department of Information Technology, Phu San Hanoi Hospital, Hanoi 100000, Vietnam; xuanbui88@gmail.com; 5 Department of Gynecological Oncology, Phu San Hanoi Hospital, Hanoi 100000, Vietnam; dr.toannguyen@hogh.vn; 6 Department of International Collaboration, Phu San Hanoi Hospital, Hanoi 100000, Vietnam; vuhienmd1511@gmail.com; 7 Independent Researcher, 36020 Solagna, Italy; cescocava@libero.it; 8 Department of Woman’s and Child’s Health, University Hospital of Padova, 35128 Padova, Italy; daniele.trevisanuto@unipd.it; 9 Emergency Care Unit, Sachs’ Children and Youth Hospital, 11883 Stockholm, Sweden; 10 Neonatal Unit, Sachs’ Children and Youth Hospital, 11883 Stockholm, Sweden; 11 Department of Clinical Science Intervention and Technology, Karolinska Institutet, 14152 Huddinge, Sweden; mats.blennow@ki.se; 12 Department of Women’s and Children’s Health, Karolinska Institutet, 17177 Solna, Sweden; 13 Department of Medical Biochemistry and Microbiology, Uppsala University, 75237 Uppsala, Sweden; 14 Intensive Care Unit and Poison Control Department, Phu San Hanoi Hospital, Hanoi 100000, Vietnam; 15 Social Work Department, Phu San Hanoi Hospital, Hanoi 100000, Vietnam; 16 University of Medicine and Pharmacy, Hanoi 100000, Vietnam; dr.duyanhnguyen@hogh.vn; 17 Board of Directors, Phu San Hanoi Hospital, Hanoi 100000, Vietnam

**Keywords:** neonatal care, incidence, intrapartum-related events, birth asphyxia, neonatal morbidity, neonatal resuscitation, Vietnam

## Abstract

Every year, 2.4 million neonates die during their first month of life and even more suffer permanent injury. The main causes are intrapartum-related events, prematurity, and infection, with sub-Saharan Africa and southern Asia being the worst affected regions. With a focus on intrapartum-related events, we aimed to assess the neonatal demographic characteristics, clinical management, and outcomes among neonates born at the largest obstetric hospital in Hanoi, Vietnam. This was a retrospective cross-sectional study that included all the inborn neonates in November 2019, which was selected as a representative month. A total of 4554 neonates were born during the study period. Of these, 1.0% (*n* = 44) were stillbirths, 0.15% (*n* = 7) died in hospital, 0.61% (*n* = 28) received positive pressure ventilation at birth, and 0.15% (*n* = 7) were diagnosed with hypoxic ischemic encephalopathy. A total of 581 (13%) neonates were admitted to the neonatal unit, among which the most common diagnoses were prematurity (37%, *n* = 217) and infection (15%, *n* = 89). Except for the intrapartum-related events, our findings are consistent with the previously documented data on neonatal morbidity. The intrapartum-related events, however, were surprisingly low in number even in comparison to high-income countries. Research on the current clinical practice at Phu San Hanoi Hospital may bring further clarity to identify the success factors.

## 1. Background

Neonatal mortality accounts for nearly half of the 5.3 million deaths that occur every year among children under five years of age [1]. The United Nations (UN) and the World Health Organization (WHO) have, in the last few years, aligned to specifically target neonatal health and end preventable deaths during the newborn period. Their aim is to reduce the overall neonatal mortality to fewer than 12 deaths per 1000 live births in all the countries of the world by 2030 [2].

Complications from prematurity, intrapartum-related events, infection, and congenital anomalies account for approximately 80% of neonatal mortality globally [1,3]. Intrapartum-related events originate from the lack of oxygen and subsequent ischemia caused by the disruption of placental blood flow, infection, or when a neonate fails to establish and maintain regular breathing at birth. The immediate damage to the brain results in hypoxic ischemic encephalopathy (HIE) and the risk of neurological sequelae or death [4]. HIE is defined as a clinical syndrome in term neonates. Hypoxic ischemic injury in preterm neonates is complex and lacks a homogenous definition [5]. HIE is diagnosed by the Thompson or Sarnat scoring systems, neuroradiology, and/or amplitude-integrated EEGs. International and local guidelines recommend initiation of hypothermic treatment within six hours from birth for neonates who meet the TOBY cooling criteria [6,7]. Intrapartum-related events are more likely to occur when the antenatal period has been complicated and in multiple or preterm births. Good quality intrapartum care has been recognized as the single most important factor in preventing intrapartum complications [8,9]. Once a neonate with respiratory depression is born, prompt drying, stimulation, and positive pressure ventilation (PPV) are the most important interventions [10]. On a global level, intrapartum-related events account for approximately 700,000 neonatal deaths every year [3]. However, neonatal mortality estimates only include live births, and it has been reported that approximately 50% of all stillbirths are due to an intrapartum-related event [11].

Prematurity-related death is mainly mediated via respiratory distress, feeding issues, and infection. The outcome of the affected neonate is greatly improved with the use of antenatal corticosteroids, kangaroo mother care, surfactant, continuous positive airway pressure (CPAP), feeding tubes, and total parenteral nutrition [12,13,14]. Infections can be congenital or acquired [15,16]. Congenital infections can be transmitted from mother to neonate during pregnancy (transplacental) or during birth (intrapartum). Neonatal sepsis can be defined as early or late onset, with early onset sepsis being more likely congenital. Early onset within the setting of a neonatal unit is defined as <72 h of life [17]. The key aspects of successfully reducing neonatal mortality secondary to infection are the identification and minimization of risk factors, supportive treatment, and adequate antibiotic/antiviral therapy. Congenital anomalies are estimated to be the fourth most common cause of neonatal death, and they contribute to a significant proportion of neonatal disease [18]. Their distribution is variable depending on both genetic and environmental factors. The rate of antenatal diagnostics and abortion highly influence the rate of congenital anomalies [19,20].

The reported rates of neonatal mortality in Vietnam are relatively good compared to other low- and middle-income countries (LMIC). The World Bank reported 10.5 deaths/1000 live births in 2019 [21]. However, a study from 2010 highlighted a substantial under-reporting of neonatal mortality in the official statistics, showing the neonatal mortality rate (16/1000) in Quang Ninh province to be fourfold higher than the rate reported to the Ministry of Health (4.2/1000) [22]. The WHO Every Newborn Action Plan (ENAP) highlights the importance of improving data collection to identify gaps in equity and quality. Its progress report in 2018 identified Vietnam as being one of a number of countries that had no perinatal death review system in place [23]. With a focus on intrapartum-related events, we aimed to assess the neonatal demographics, clinical management, and outcomes among neonates born at the largest obstetric hospital in Hanoi, the capital of Vietnam. The results of this study should raise awareness regarding the main causes of neonatal mortality and morbidity in urban Vietnam and how they compare to the primary causes on a global scale.

## 2. Methods

### 2.1. Study Setting

With approximately 40,000 births annually, Phu San Hanoi Hospital is the largest obstetric hospital in Hanoi and the top government public hospital in Vietnam. It is situated next to Vietnam National Children’s Hospital (VNCH). The hospital’s overall c-section rate is greater than 50%, approximately half of which are emergency c-sections. Most newborn neonates are admitted to the postnatal ward for 24 h before being discharged. Neonates who are born with clinical distress and/or risk factors are admitted to the neonatal unit, which is staffed by 72 nurses and 21 doctors. The neonatal unit at Phu San Hanoi Hospital is divided into a neonatal intensive care unit (NICU; 38 beds and 29 incubators) and a high dependency unit (50 beds).

Many structural and clinical changes have taken place at Phu San Hanoi Hospital during the past few years. Birth attendants who assisted 4–6 birthing women simultaneously 5 years ago now assist 1–2 simultaneous vaginal deliveries. The following therapies are currently available at Phu San Hospital: mechanical ventilation (available since 1996), surfactant therapy (2000), CPAP (2001), high frequency oscillatory ventilation (2012), umbilical venous catheterization (2012), umbilical arterial catheterization (2015), and minimally invasive surfactant administration techniques (Insure 2016, Lisa 2018). Admission criteria to the neonatal unit include: preterm birth <34 weeks, low birth weight <2000 g, neonates with ≥2 risk factors (maternal fever, stained or foul-smelling amniotic fluid, prolonged rupture of membranes ≥18 h, premature rupture of membranes, maternal colonization by group B streptococcus), maternal insulin dependent diabetes mellitus, need for PPV at birth, respiratory failure, and congenital anomalies.

Neonates who require surgery (including severe pneumothorax), blood exchange transfusion, cooling or ECMO, or suffer from severe pulmonary hypertension, multi-resistant bacteremia, congenital metabolic disorders, or, rarely, on family request, are transferred to the neighboring hospital’s neonatal unit at VNCH.

### 2.2. Study Design

This was a retrospective cross-sectional study including all the inborn neonates at Phu San Hanoi Hospital in November 2019. The number of livebirths, stillbirths, and neonatal unit admissions was identified using the local electronic system. Liveborn neonates deceased prior to admission to the neonatal unit were identified from the local mortality book. Data related to the neonates admitted to the neonatal unit were extracted from the neonates’ medical files by two neonatologists using a standardized hard copy case report form (CRF). The variables of interest included: maternal and study subject demographics, perinatal risk factors, medical diagnoses, clinical management, and outcome. Data were transferred from the CRFs into an electronic database (RedCap, Vanderbilt, TN, USA) by two non-clinical data officers. Before data transfer, the officers completed a one-day orientation and training that focused on data entry into RedCap from clinical CRFs. For quality control purposes, 5% of the transferred data were double checked by the project coordinator.

The definitions used include: prematurity <37 weeks gestation, hypoglycemia <2.6 mmol/L, hypothermia <36 °C, and hyperbilirubinemia >120 mmol/L (>7 mg/dL). Bronchopulmonary dysplasia was defined as oxygen dependency at 36 weeks’ gestation (for neonates <32 weeks), or as oxygen dependency at 29–55 days of life (for neonates ≥32 weeks). Respiratory distress syndrome (RDS) was diagnosed in preterm neonates who required supplemental oxygen, CPAP, or mechanical ventilation, and when the respiratory distress could not be explained by another diagnosis. Surfactant was administered based on the European Consensus Guidelines on the Management of RDS–2019 [24]. HIE was diagnosed in term as well as preterm neonates based on clinical exam by the Sarnat scoring system in neonates that fulfilled one of the asphyxia criteria: Apgar score <5 at 10 min of life, pH < 7.0 within the first hour of life, or base excess < −16 mmol/L within the first hour of life. Both confirmed (culture positive) and suspected infections (based on risk factors, clinical exam, blood tests, and radiology) were recorded. Infection was defined as congenital if an identified pathogen was known to be vertically transmitted (e.g., TORCH infection) or if the infection was diagnosed within the first 72 h of life. We defined metabolic disease and tumors as congenital anomalies according to the WHO’s definition (a structural or functional anomaly that occurs during intrauterine life) [18]. The indication for antibiotic administration was determined based on a hierarchal level where the highest valid cause was documented (culture confirmed > elevated infectious markers and/or suspicious chest X-ray > known risk factors/suspicious clinical exam > unknown).

### 2.3. Ethical Approval

The study was approved by the Vietnamese Institutional Review Board in Human Research Dinh Tien Hoang Institute of Medicine (IRB-2001) on 26 March 2020. The data analysis took place in Sweden and was approved by the Swedish Ethical Review Authority (Dnr 2021-00064) on 5 March 2021.

### 2.4. Statistical Analysis

Variables were reported as frequencies and percentages for categorical data, and medians with interquartile ranges (IQR) for continuous data. The variables were compared among birthweight categories using a chi-square test and Fisher’s exact test (categorical variables), and Kruskal–Wallis (continuous variables). A *p*-value less than 0.05 was considered significant. Data analysis was performed using STATA version 16.1 (StataCorp LCC, College Station, TX, USA).

## 3. Results

**Figure 1:**
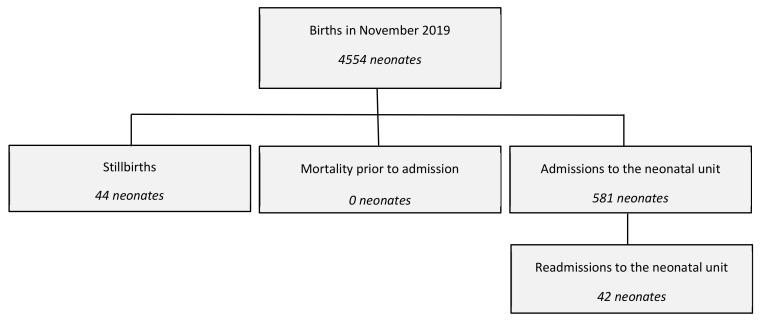
Flow chart of neonates included in the study.

A total of 4554 neonates were born at the hospital in November 2019. Of these, 1.0% were stillbirths (*n* = 44) and 13% (*n* = 581) were admitted to the neonatal unit. There were no deaths of liveborn neonates prior to neonatal unit admission (Figure 1).

### 3.1. Demography, Mode of Delivery, and Resuscitation of Neonates Admitted to the Neonatal Unit

The full demographic details are presented in Table 1. Of the neonates admitted to the neonatal unit, 37% were preterm, 2.9% were extremely preterm (<28 weeks’ gestation), and 1.9% had an extremely low birth weight (ELBW, <1000 g). Approximately 60% were delivered by c-section, and only one neonate had an instrumental delivery (forceps). At birth, the overall PPV rate was 0.6% (*n* = 28/4554 births), and, among the neonates admitted to the neonatal unit, 4.8%. The overall incidence of endotracheal intubations (ETI) was 0.22% (10/4554 births), and the proportion of intubation among the neonates receiving PPV was 36% (10/28 neonates). The neonates with a lower birth weight had a significantly higher incidence of 5-min Apgar <7. This group also required significantly more PPV, including PPV delivered via ETI.

### 3.2. Diagnoses in Neonates Admitted to Neonatal Unit

Table 2 reports the diagnoses for the neonates admitted to the neonatal unit, both overall and in relation to birth weight. Consistent with the need for PPV, HIE was more common in the very low birth weight (VLBW) neonates (7.0%, *n* = 4) compared to low birth weight (LBW) neonates (1.2%, *n* = 2) and normal birth weight (NBW) neonates (0.3%, *n* = 1). Only one neonate was diagnosed with seizures, which appears under “other” diagnoses.

### 3.3. Clinical Management of Neonates Admitted to the Neonatal Unit

The most common therapies at the neonatal unit were: use of a feeding tube (54%, *n* = 313), phototherapy (31%, *n* = 180), antibiotic administration (24%, *n* = 140), and oxygen supplementation (23%, *n* = 134) (Table 3). One neonate was transferred to VNCH for therapeutic hypothermia. All the ELBW neonates who survived their first day of life (*n* = 10) received antibiotics, total parenteral nutrition, and caffeine. Ninety percent of the ELBW neonates received non-invasive respiratory support (CPAP). Less than one-third of all the neonates with RDS received surfactant (*n* = 31). Eight neonates received surfactant without ever requiring mechanical/oscillatory ventilation.

### 3.4. Outcome of Neonates Admitted to the Neonatal Unit

The outcomes are presented in Table 4. The overall mortality rate at Phu San neonatal unit (excluding neonates discharged to VNCH or other neonatal units) was 0.2% (*n* = 7), of whom five had a birth weight <1000 g, one died from a congenital diaphragmatic hernia, and one died from complications due to metabolic disease. Just over half (53%, *n* = 310) of the neonates admitted to the neonatal unit were discharged within 24 h of admission, with 6.7% (*n* = 39) being transferred to VNCH or another hospital. A total of 13% (*n* = 73) of the admitted neonates were transferred to VNCH or another hospital. Amongst the neonates not discharged within their first 24 h, the median duration of admission was 6 (IQR 3-13) days, with the lower birth weight neonates having a significantly longer stay. A total of 6.9% (*n* = 40) were readmitted after discharge, with phototherapy (90%, *n* = 36) being the most common indication for readmission.

### 3.5. Quality Control

We found a 0.6% mismatch between the hard copy CRFs and the RedCap insertions.

## 4. Discussion

This study showed a lower-than-expected rate of intrapartum-related events at Phu San Hanoi Hospital, whereas the main causes of neonatal morbidity and mortality otherwise mirrored those causes on a global scale. The overall PPV rate was 0.61%, and approximately one-third of those patients progressed to ETIs. A global review from 2011 estimated that 3 to 6% of newborn neonates require PPV at birth, and <1% require ETI [25]. A study from the United States including neonates with a gestational age ≥ 34 weeks found that 6% required PPV at birth, and, of these, 22% received ETI [26]. Two Norwegian studies reported an overall PPV rate of 3% and 4%, respectively, with ETI requirements of 9% and 6.9%. The latter showed a higher PPV rate in neonates <34 weeks (21%) compared to >34 weeks (3%) [27,28]. A Brazilian study that looked at neonates with NBW without a congenital anomaly, reported a PPV rate of 3.1% [29]. Research conducted at a hospital in Tanzania showed that 6.5% of the newborn neonates needed PPV at birth [30]. Finally, research conducted in Uganda by part of our own research team found that 8.6% of the included neonates (gestational age ≥34 weeks and birth weight ≥2000 g) required PPV at birth [31].

The incidence of HIE was 0.15% (assuming that no neonates who transferred to VNCH on another indication received a subsequent HIE comorbidity diagnosis). The neonates at Phu San’s maternity hospital who require cooling therapy are transferred to VNCH. Only one neonate who received PPV was transferred during the time period when cooling was still indicated (within six hours from birth). We confirmed that this neonate did indeed receive therapeutic hypothermia, which accounts for 0.022% of the total number of neonates born during the study period. As described above, it is difficult to know the true incidence of HIE. The original definition only included term neonates. However, the understanding has lately moved towards also including preterm neonates, even if a homogenous definition is still lacking [5]. We have decided to report HIE among all neonates. A systematic review, mostly including HIC and a mix of studies including term and/or preterm neonates, estimated the range to be between 0.1 and 0.8% [32]. One study based in Uganda that included term neonates with a birth weight >2000 g reported an HIE incidence of 3.6% [33]. Another study based in Tanzania that included all neonates reported an HIE incidence of 10.7% [34].

Despite the difficulty of defining intrapartum-related events, the above data indicate a low rate of these events at Phu San Hanoi Hospital. This finding is difficult to interpret. We will discuss some possible explanations. We have noted a consistently high c-section rate, over 50% of all births. Previous studies have reported a directly increased mortality (neonatal and maternal) if the c-section rate is below 5%, and a possible beneficial effect in c-section rates up to 10 to 15% [35,36,37]. The WHO stated in 2016 that, due to a lack of data, it was not possible to draw conclusions on the effect on stillbirths and perinatal morbidity when c-section rates exceeded 10% [35]. A high c-section rate in Vietnam has been reported previously. A study in 2014 looked at 1350 women using a representative sample of households at a national level and found that urban areas had nearly twice the c-section rate (42%) compared to rural (23%) [38]. A study from Da Nang, the third largest city in Vietnam, reported a c-section rate of 58% in public hospitals and 70% in private hospitals [39]. Another study from Nha Trang, a tourist city in southern Vietnam, reported a c-section rate of 44% [40]. The high proportion of emergency c-sections at Phu San Hanoi Hospital might indicate a lower-than-average threshold for converting an unsatisfactory vaginal delivery progress to c-section. We report that only one neonate was assisted by instrumental delivery (forceps). Previous studies have reported an instrumental delivery rate between 5 and 20%, with a failure rate of 5 to 10% [41]. An indirect measure of good obstetric care is the stillbirth rate. We report a stillbirth rate of 1.0%. The average stillbirth rate in an HIC has previously been reported to be 0.3%, and the stillbirth rate in sub-Saharan Africa and southern Asia has been estimated to approximate 3% [15]. Moreover, proper antenatal care can contribute to fewer intrapartum-related events. One study reported that the average number of antenatal care visits for women in Vietnam was 7.7 visits in urban areas and 4.4 visits in rural areas [42]. The WHO recently recommended a minimum of eight antenatal visits [43]. We did not report the number of antenatal care visits in our study as this information was not retrospectively available.

We found an 8% prematurity rate. The WHO reported in 2018 a prematurity rate ranging between 5% and 18% [44]. The most common complications among our preterm neonates admitted to the neonatal unit include respiratory distress, feeding difficulties, jaundice, and hospital-acquired infection. These mirror complications that are commonly reported in preterm neonates in both HIC and LIC [13,14,44,45,46]. Neonates with a birth weight <1000 g had a survival rate of 55%, which is more similar to previous reports from northern India (52%) than reports from Japan (70%) and Hong Kong (79%) [47,48,49].

The overall hospital rate of congenital malformations was 1.5% in inborn neonates. A recent study in Da Nang, an urban hub in central Vietnam, reported a congenital anomaly rate of 3.8%. Even though this rate is somewhat higher than what we report, the distribution was similar, with approximately 50% of the anomalies affecting the cardiovascular system [50]. The actual incidence at Phu San could be higher if some affected neonates were initially missed, as any malformation is an indication for neonatal unit admission at Phu San. A large study in south India, of 36,074 neonates over 10 years, found an incidence of malformation of 1.3%, with the majority being musculoskeletal (affecting bone and muscle development in the skull, trunk, or limbs, where congenital talipes equinovarus comprised 74% of the musculoskeletal group). The same study reported congenital anomalies in 9.8% of the stillbirths [51]. We did not specifically assess stillbirths in relation to congenital anomalies at Phu San.

We report a culture-confirmed nosocomial infection rate of 2.8% among the neonates admitted to the neonatal unit. When suspected infections (based on risk factors, clinical exam, blood tests, and radiology) are included, the rate increases to 8.1%. Previous studies in the US and Taiwan have reported a nosocomial infection rate around 11% within the neonatal unit [52,53]. As opposed to nosocomial infection rates, the congenital infection rate should not be affected by neonatal transfer. We found culture- or radiology-verified suspected congenital infection in 0.15% of the neonates born during the study period. This is similar to previous reports from within the USA [17]. The TORCH infection rate was 0.13% of the births. These infections are known to increase incidences of LBW, congenital anomalies, and stillbirths. Its rate varies among countries and is highly dependent on implemented screening programs [54].

Finally, our observed mortality rate was 0.15%. However, the actual mortality rate depends on the mortality rate of the transferred neonates and the 20 neonates for whom the final discharge location is unknown. Thus, our actual mortality rate lies between 0.15 and 2.2%. We assume, however, that a minority of the transferred and unknown discharge location neonates died, and, therefore, the true figure to be closer to the one we report. Any future study should include neonatal follow-up at VNCH.

## 5. Limitations

Our study has a number of limitations. Its retrospective design carries the limitations of bias always associated with such studies. The cross-sectional period was restricted to one month, which may not mirror the seasonal variation, although the month was carefully selected to be as representative as possible. The sickest neonates at Phu San neonatal unit are transferred to another hospital, which has likely affected our reported infection and mortality rates. This differs from the data presented on intrapartum-related events, prematurity, and congenital anomalies, where it would be difficult to see any transfer-related bias. Finally, as we only looked at the hard copy medical records for the neonates who were admitted to the neonatal unit, there could be a certain degree of underreporting here (in case a neonate was not admitted, for some reason, despite having an admission indication).

## 6. Conclusions

The main local causes of neonatal morbidity and mortality at Phu San Hanoi Hospital were prematurity, infection, congenital malformation, and intrapartum-related events. This is consistent with the leading causes of neonatal adverse outcomes on a global scale. The intrapartum-related events, however, were found to be surprisingly low even in comparison to HIC. A prospective study focusing on intrapartum-related events and immediate resuscitation is necessary for confirming the findings of this study. If a future study can confirm the low rate of intrapartum-related events, attempts should be made to describe the perinatal care at Phu San Hanoi Hospital. Addressing intrapartum-related events and learning how to prevent them will benefit neonates and their families globally.

## Figures and Tables

**Table 1:** Demography, mode of delivery, and resuscitation of neonates admitted to the neonatal unit at Phu San Hospital in November 2019.

	All Neonates *N* = 581		Birth Weight ^a^ <1500 g *N* = 57		Birth Weight ^a^ 1500–2500 g *N* = 167		Birth Weight ^a^ >2500 g *N* = 353	
	*n*/Median	%/IQR	*n*/Median	%/IQR	*n*/Median	%/IQR	*n*/Median	%/IQR
**General**								
Female	256	44.1	24	42.1	83	49.7	148	41.9
Multiple births	101	17.4	23	40.4	55	32.9	22	6.2
**Maternal factors**						0.0		
Age (years)	29	26–33	28	26–31	29	26–33	29	26–33
Primipara	270	46.5	35	61.4	83	49.7	151	42.8
Previous preterm births	14	2.4	0	0.0	3	1.8	11	3.1
Previous miscarriage/stillbirth	78	13.4	6	10.5	21	12.6	51	14.4
**Perinatal factors**								
Stained/foul-smelling meconium	22	3.8	1	1.8	7	4.2	14	4.0
PROM ^b^	95	16.4	14	24.6	41	24.6	41	11.6
Maternal infection during labour	6	1.0	2	3.5	2	1.2	2	0.6
Maternal temp. <36°C or >38 °C	2	0.3	0	0.0	0	0.0	2	0.6
Preeclampsia	26	4.5	4	7.0	16	9.6	6	1.7
Eclampsia	1	0.0	0	0.0	0	0.0	1	0.0
Placenta praevia	16	2.8	3	5.3	3	1.8	10	2.8
Placenta abruption	4	0.7	1	1.8	0	0.0	3	0.8
**Mode of delivery**								
Spontaneous vaginal delivery	233	40.1	37	64.9	62	37.1	133	37.7
Assisted (forceps or vacuum)	1	0.2	0	0.0	0	0.0	1	0.3
Elective c-section	225	38.7	9	15.8	57	34.1	159	45.0
Emergency c-section	119	20.5	11	19.3	48	28.7	60	17.0
**Gestational age (weeks’) ^c^**								
<28	17	2.9	17	29.8	0	0.0	0	0.0
28–32	32	5.5	23	40.4	9	5.4	0	0.0
32–37	168	28.9	16	28.1	111	66.5	41	11.6
>37	359	61.8	1	1.8	46	27.5	311	88.1
**Resuscitation**								
Apgar score at 1 min	8	7–8	6	5–6.5	7	7–8	8	8–8
Apgar score at 5 min	9	8–9	7	6–7.5	8	8–9	9	9–9
Apgar score <7 at 5 min	30	5.2	22	38.6	3	1.8	4	1.1
Face mask ventilation ^d^	26	4.5	15	26.3	9	5.4	2	0.6
Endotracheal intubation	10	1.7	3	5.3	4	2.4	3	0.8

^a^ Four neonates missing. ^b^ Combines preterm and term premature rupture of membrane (PROM). ^c^ Five neonates missing. ^d^ Includes eight neonates who also received endotracheal intubation.

**Table 2:** Diagnoses in all neonates admitted to the neonatal unit (first admission) at Phu San Hospital in November 2019.

	All Neonates *N* = 581		Birth Weight ^a^ <1500 g *N* = 57		Birth Weight ^a^ 1500–2500 g *N* = 167		Birth Weight ^a^ >2500 g *N* = 353	
	*n*	% ^b^	*n*	% ^b^	*n*	% ^b^	*n*	% ^b^
**Birth weight and gestational age**								
Prematurity	217	37.3	56	98.2	120	71.9	41	11.6
Small for gestational age	44	7.6	12	21.1	30	18.0	2	0.6
Large for gestational age	4	0.7	0	0.0	1	0.6	3	0.8
**Cardiopulmonary**								
Respiratory distress syndrome	96	16.5	47	82.5	33	19.8	15	4.2
Respiratory distress resolved within 6 h	60	10.3	6	10.5	33	19.8	21	5.9
Transient tachypnoea of the newborn	23	4.0	0	0.0	6	3.6	17	4.8
Cyanotic attack	23	4.0	0	0.0	2	1.2	21	5.9
Patent ductus arteriosus	11	1.9	8	14.0	3	1.8	0	0.0
Apnea requiring treatment	8	1.4	6	10.5	2	1.2	0	0.0
Bronchopulmonary dysplasia	4	0.7	4	7.0	0	0.0	0	0.0
Arrhythmia ^c^	4	0.7	0	0.0	0	0.0	4	1.1
Persistent pulmonary hypertension	3	0.5	0	0.0	0	0.0	3	0.8
Pleural effusion	3	0.5	0	0.0	2	1.2	1	0.3
**Hospital acquired infection**								
All combined	47	8.1	39	68.4	5	3.0	3	0.8
Diagnosis:								
Suspected based on other parameters	23	4.0	16	28.1	4	2.4	3	0.8
Culture confirmed	16	2.8	16	28.1	0	0.0	0	0.0
Chest X-ray confirmed pneumonia	8	1.4	7	12.3	1	0.6	0	0.0
**Congenital infection**								
All combined	42	7.2	5	8.8	12	7.2	25	7.1
High risk based on other parameters	19	3.3	2	3.5	3	1.8	14	4.0
TORCH infection	6	1.0	1	1.8	3	1.8	2	0.6
Maternal syphilis or HIV	5	0.9	0	0.0	1	0.6	4	1.1
Prolonged ROM	5	0.9	0	0.0	4	2.4	1	0.3
Culture confirmed or chest X-ray verified	7	1.2	2	3.5	1	0.6	4	1.1
**Intrapartum-related event**								
PPV requirement	28	4.8	15	26.3	9	5.4	4	1.1
Hypoxic ischaemic encephalopathy	7	1.2	4	7.0	2	1.2	1	0.3
Birth related trauma ^d^	4	0.7	0	0.0	1	0.6	3	0.8
**Gastrointestinal**								
Discolored vomiting	26	4.5	1	1.8	0	0.0	25	7.1
Other minor disturbance	6	1.0	0	0.0	0	0.0	6	1.7
Necrotizing enterocolitis or obstruction	5	0.9	1	1.8	1	0.6	3	0.8
**Other**								
Jaundice	184	31.7	36	63.2	64	38.3	83	23.5
Maternal diabetes mellitus	78	13.4	0	0.0	13	7.8	65	18.4
Congenital anomaly ^e^	67	11.5	3	5.3	13	7.8	51	14.4
Other ^f^	30	5.2	2	3.5	8	4.8	20	5.7
Intraventricular hemorrhage	3	0.5	3	5.3	0	0.0	0	0.0

^a^ Four neonates missing. ^b^ Some neonates have more than one diagnosis. Proportions (%) are presented in reference to the number of neonates. ^c^ Includes: bradycardia (*n* = 3) and extrasystole (*n* = 1). ^d^ Includes: clavicular fracture (*n* = 2); facial nerve injury following forceps usage (*n* = 1); large cephalohematoma (*n* = 1). ^e^ Includes: cardiovascular (*n* = 32); gastrointestinal (*n* = 10); pulmonary (*n* = 9); suspected or confirmed syndrome (*n* = 5); dysmelia (*n* = 2); renal (*n* = 1); neurological (*n* = 1); metabolic (*n* = 1); tumour (*n* = 1); unknown (*n* = 5). ^f^ Includes all the conditions for which *n* ≤ 2 or diagnoses that are no longer indication for admission.

**Table 3:** Clinical management of all neonates admitted to the neonatal unit (first admission) at Phu San Hospital in November 2019.

	All Neonates *N* = 581		Birth Weight ^a^ <1500 g *N* = 57		Birth Weight ^a^ 1500–2500 g *N* = 167		Birth Weight ^a^ >2500 g *N* = 353	
	*n*/Median	%/IQR	*n*/Median	%/IQR	*n*/Median	%/IQR	*n*/Median	%/IQR
**Antibiotics**								
All causes combined	140	24.1	50	87.7	43	25.7	46	13.0
Indication:								
Based on clinical risk factors	91	15.7	22	38.6	37	22.2	31	8.8
Based on blood tests ^b^ and radiology	31	5.3	11	19.3	6	3.6	14	4.0
Culture-confirmed	17	2.9	16	28.1	0	0.0	1	0.3
Unknown	1	0.2	1	1.8	0	0.0	0	0.0
**Respiratory**								
Oxygen therapy	134	23.1	41	71.9	55	32.9	37	10.5
CPAP	58	10.0	33	57.9	18	10.8	6	1.7
Mechanical ventilation	46	7.9	18	31.6	10	6.0	17	4.8
Oscillatory ventilation ^c^	9	1.5	6	10.5	1	0.6	2	0.6
Duration of ventilation (days)	4	2–9	9	7–14	4	2–4	2	1-2
Caffeine administration	68	11.7	44	77.2	22	13.2	0	0.0
Surfactant administration	31	5.3	17	29.8	11	6.6	2	0.6
**Fluids, electrolytes, and nutrition**								
Feeding tube	313	53.9	48	84.2	52	31.1	30	8.5
Glucose infusion	82	14.1	8	14.0	34	20.4	40	11.3
Total parenteral nutrition	58	10.0	44	77.2	11	6.6	2	0.6
Weight documented at discharge	168	28.9	40	70.2	57	34.1	69	19.5
**General**								
Analgesia administration	16	2.8	3	5.3	6	3.6	7	2.0
Phototherapy	180	31.0	40	70.2	59	35.3	81	22.9
Inotropic drugs administration	12	2.1	7	12.3	1	0.6	4	1.1
Gastric lavage	3	0.5	0	0.0	0	0.0	3	0.8
Otrivin administration	1	0.2	0	0.0	0	0.0	1	0.3

^a^ Four neonates missing. ^b^ Excludes blood culture-confirmed infection. ^c^ All nine neonates also received mechanical ventilation.

**Table 4:** Clinical outcomes among all neonates admitted to the neonatal unit at Phu San Hospital in November 2019.

	All Neonates *N* = 581		Birth Weight ^a^ <1500 g *N* = 57		Birth Weight ^a^ 1500–2500 g *N* = 167		Birth Weight ^a^ >2500 g *N* = 353	
	*n*/Median	%/IQR	*n*/Median	%/IQR	*n*/Median	%/IQR	*n*/Median	%/IQR
**First admission**								
Number of neonates	581	100.0	57	100.0	167	100.0	353	100.0
Duration <1 day	310	53.4	4	7.0	76	45.8	230	65.0
If >1 day, total duration (days) ^b^	6	3–13	35	21–45	7	5–11	3	2–5
**Readmission**								
Number of neonates	40	6.9	1	1.8	19	11.4	20	5.6
Number of readmissions	42 ^c^	-	1	-	21	-	20	-
Duration <1 day	7	17.5	0	0.0	4	15.8	4	20.0
If >1 day, total duration (days)	4	3–4	3	3–3	3	3–4	3	2–4
Phototherapy	36	90.0	1	100.0	18	94.7	17	85.0
Antibiotic therapy	3	7.5	0	0.0	2	10.5	1	5.0
Feeding tube	2	5.0	0	0.0	2	10.5	0	0.0
Glucose infusion	1	2.5	0	0.0	0	0.0	1	5.0
Oxygen therapy	1	2.5	0	0.0	1	5.3	0	0.0
**Quality of care parameters**								
Number of admissions	623	100.0	58	100.0	187	100.0	374	100.0
Documentation of vital parameters ^d^	603	96.8	56	96.6	186	99.5	361	96.5
Initial hypothermia	14	2.3	9	16.1	1	0.5	4	1.1
Improved to >36 °C within 2 h	8	57.1	7	77.8	0	0.0	1	25.0
Documentation of blood glucose ^d^	358	57.5	47	81.0	133	71.1	178	47.6
Initial hypoglycemia	41	11.5	7	14.9	18	13.5	16	9.0
Improved to >2.6 mmol/L within 30 min	37	90.2	7	100.0	15	83.3	15	93.8
**Final discharge location ^e^**								
Home	221	38.0	30	52.6	80	48.2	110	31.1
Postnatal ward Phu San	267	46.0	3	5.3	69	41.6	195	55.1
VNCH	49	8.4	11	19.3	12	7.2	26	7.3
Other hospital	24	4.1	4	7.0	3	1.8	17	4.8
If discharged to VNCH/other hospital,								
discharged within 24 h ^f^	39	53.4	0	0.0	4	26.7	35	81.4
**Mortality**								
Deaths	7	1.2	5	8.8	0	0.0	2	0.6
Timing (days of life)	7	1–20	10	7–20	-	-	3	1–5

^a^ Four neonates missing. ^b^ Seven neonates missing. ^c^ One neonate was readmitted three times. ^d^ Documentation within 30 min of admission (vital parameters = heart rate, SpO_2_, and body temperature). ^e^ Twenty neonates missing. ^f^ Three neonates missing.

## Data Availability

The data presented in this study are available on request from the corresponding author. The data are not publicly available due to a lack of specification in the ethical application and approval.

## Abbreviations

CRF	case report form
ETI	endotracheal tube intubation
PPV	positive pressure ventilation
HIE	hypoxic ischemic encephalopathy
HIC	high income countries
LIC	low income countries
LMIC	low-middle income countries
UN	United Nations
WHO	World Health Organization
VNCH	Vietnam National Children’s Hospital
NBW	normal birth weight
LBW	low birth weight
VLBW	very low birth weight
ELBW	extremely low birth weight
RDS	respiratory distress syndrome
PROM	prolonged (≥18 h) rupture of membranes
